# A CRISPR/Cas9-based genome-editing platform enabling efficient and precise gene replacement in *Lipomyces starkeyi*

**DOI:** 10.1093/femsyr/foag014

**Published:** 2026-04-23

**Authors:** Rikako Sato, Kaito Maruyama, Satoshi Ara, Masayuki Shibata, Yosuke Shida, Wataru Ogasawara, Harutake Yamazaki, Hiroaki Takaku

**Affiliations:** Department of Applied Life Sciences, Niigata University of Pharmacy and Medical and Life Sciences, Akiha-ku, Niigata 956-8603, Japan; Department of Applied Life Sciences, Niigata University of Pharmacy and Medical and Life Sciences, Akiha-ku, Niigata 956-8603, Japan; Research Institute for Creating the Future, FUJI OIL Co., Ltd, Tsukubamirai-shi, Ibaraki 300-2497, Japan; Research Institute for Creating the Future, FUJI OIL Co., Ltd, Tsukubamirai-shi, Ibaraki 300-2497, Japan; Department of Bioengineering, Nagaoka University of Technology, Nagaoka, Niigata 940-2188, Japan; Department of Bioengineering, Nagaoka University of Technology, Nagaoka, Niigata 940-2188, Japan; Department of Applied Life Sciences, Niigata University of Pharmacy and Medical and Life Sciences, Akiha-ku, Niigata 956-8603, Japan; Department of Applied Life Sciences, Niigata University of Pharmacy and Medical and Life Sciences, Akiha-ku, Niigata 956-8603, Japan

**Keywords:** *Lipomyces starkeyi*, CRISPR/Cas9, oleaginous yeast, genome editing, homologous recombination, nonhomologous end joining

## Abstract

*Lipomyces starkeyi* is a promising oleaginous yeast with industrial potential. However, its genome engineering remains constrained by low gene-targeting efficiency and the requirement for long homologous regions. Herein, we established a CRISPR/Cas9 genome-editing platform for *L. starkeyi* by expressing codon-optimized *Streptococcus pyogenes Cas9* fused to an SV40 nuclear localization signal. Furthermore, *in vitro*-transcribed single-guide RNAs (sgRNAs) were directly delivered into the host, eliminating the need for endogenous RNA polymerase III–dependent sgRNA expression. CRISPR/Cas9 activity was validated using a codon-optimized *Aequorea coerulescens* GFP reporter. Cas9-induced frameshift mutations caused *GFP* disruption, leading to fluorescence loss. Gene replacement at the *LsURA3* locus was evaluated using donor constructs with homologous regions ranging from 50–3000 bp. In a Cas9-expressing wild-type background, precise gene replacement was dependent on homology arm length, increasing from 36% with 50-bp arms to 80% with 3000-bp arms. Notably, in a Cas9-expressing Δ*lslig4* strain with suppressed non-homologous end joining (NHEJ), precise gene replacement was achieved with 100% accuracy using 50-bp homology arms under CRISPR/Cas9-dependent conditions. Together, these results demonstrate that a Pol III-independent CRISPR/Cas9 system combined with NHEJ suppression enables precise genome editing in *L. starkeyi*, providing a foundation for functional genomics and metabolic engineering.

## Teaser

A CRISPR/Cas9 genome-editing platform in *Lipomyces starkeyi* enables efficient and precise gene replacement with minimal homology by combining Cas9-induced double-strand breaks and suppression of non-homologous end joining via LsLIG4 deletion.

## Introduction

Oleaginous yeasts have attracted attention as alternative microbial platforms for the sustainable production of lipids and lipid-derived chemicals. These micro-organisms can accumulate large quantities of triacylglycerols (TAGs), which serve as precursors for biofuels, oleochemicals, and nutraceuticals. Among all the documented oleaginous yeast species, *Lipomyces starkeyi* is particularly notable due to its exceptional lipid accumulation capacity, which can reach up to 85% of its dry cell weight under optimized conditions (Takaku et al. 2020a, Juanssilfero et al. 2018). Besides glucose, *L. starkeyi* can metabolize various other carbon sources, including xylose, arabinose, cellobiose, maltose, sucrose, starch, and glycerol (Takaku et al. 2020a, Oguri et al. 2012). Furthermore, unlike other well-studied oleaginous yeasts, such as *Yarrowia lipolytica* and *Rhodosporidium toruloides, L. starkeyi* secretes an α-amylase (LsAmy1p) and two α-glucosidases (LsAgd1p and LsAgd2p), enabling the direct degradation and assimilation of starch (Mine et al. 2025). Therefore, due to its metabolic versatility and unique starch-degrading capabilities, *L. starkeyi* offers significant advantages for several industrial applications, including the conversion of lignocellulosic hydrolysates and agro-industrial residues, as well as starch-based bioprocesses.

Several molecular tools and transformation methods have been established to develop *L. starkeyi* as a microbial cell factory. Early studies introduced strong promoters and selectable markers (Calvey et al. 2014), followed by the development of multiple transformation strategies, including lithium acetate-mediated transformation (Calvey et al. 2014), polyethylene glycol (PEG)-mediated spheroplast transformation (Oguro et al. 2015), *Agrobacterium tumefaciens*-mediated transformation (Lin et al. 2017), and electroporation (Takaku et al. 2020b). Furthermore, a multicopy integration system targeting the 18S rRNA gene locus has been reported (Oguro et al. 2015). Despite these advances, precise genome editing in *L. starkeyi* remains challenging.

In *L. starkeyi*, non-homologous end joining (NHEJ) predominates for DNA double-strand break (DSB) repair, leading to inefficient and frequently inaccurate integration of exogenous DNA fragments. This low efficiency of precise gene targeting represents a major limitation in the genetic engineering of *L. starkeyi*. DNA ligase IV is a key component of the NHEJ pathway and catalyzes the final ligation step during repair of DSBs. In several yeasts and fungi, disruption of *LIG4* has been shown to reduce NHEJ activity and enhance homologous recombination (HR)-mediated gene targeting. Although disruption of *LsLIG4*, encoding DNA ligase IV, significantly improved homologous recombination (HR)-mediated gene targeting, reliable genome modification still requires long homology arms, representing another key bottleneck in *L. starkeyi* genetic engineering (Oguro et al. 2017). Similar challenges have also been reported for other nonconventional yeasts, where low HR efficiency and limited genetic toolkits constrain precise genome manipulation (Cai et al. 2019, Park et al. 2022).

The clustered regularly interspaced short palindromic repeats (CRISPR) and CRISPR-associated protein (Cas) system, originally identified as an adaptive immune mechanism in bacteria and archaea (Barrangou et al. 2007), has revolutionized genome engineering due to its simplicity, efficiency, and versatility. The CRISPR/Cas9 system requires only a Cas9 endonuclease and a single-guide RNA (sgRNA), which directs Cas9 to a specific genomic locus and induces a targeted DSB. Subsequent repair by NHEJ results in small insertions or deletions, whereas repair by HR using a donor template enables precise genome modification (Donohoue et al. 2018, Cai et al. 2019, Xia et al. 2023).

While *Saccharomyces cerevisiae* remains the most extensively characterized yeast for CRISPR-based genome editing (Rainha et al. 2021), nonconventional yeasts are being increasingly used for CRISPR technologies. In *Y. lipolytica*, codon-optimized Cas9 together with synthetic RNA polymerase III promoters was used to achieve efficient gene disruption and metabolic engineering (Schwartz et al. 2016), leading to the development of CRISPR-Cas12a/Cpf1 systems that further expanded editing capabilities (Larroude et al. 2020, Yang et al. 2020). In *R. toruloides*, genes involved in carotenoid biosynthesis and central metabolism were successfully edited using CRISPR/Cas9-mediated mutagenesis and donor-assisted HR (Rodriguez et al. 2016, Jiao et al. 2019). These studies demonstrate the broad applicability of CRISPR-based systems in oleaginous yeasts.

Despite these advances, the application of CRISPR/Cas systems to *L. starkeyi* has remained limited, mainly due to its strong reliance on NHEJ and the lack of validated genetic elements for efficient sgRNA expression. This compromises the efficiency of precise genome editing, underscoring the need for CRISPR tools specifically optimized for this species.

In this study, we established a CRISPR/Cas9-based genome-editing platform tailored to *L. starkeyi*. By constitutively expressing a nuclear-localized, codon-optimized Cas9 and directly delivering *in vitro*-transcribed sgRNAs, we circumvented the need for endogenous RNA polymerase III promoters. Using a *GFP* disruption assay, we validated robust Cas9 activity *in vivo*, which resulted in frameshift mutations and rapid loss of GFP fluorescence. This confirmed that Cas9-mediated gene disruption was successful in *L. starkeyi*. Additionally, we observed that CRISPR/Cas9-induced DSBs significantly enhanced HR efficiency at the *LsURA3* locus. Using a combination of CRISPR/Cas9 and *LsLIG4* deletion, we achieved highly efficient and precise gene replacement with homology arms as short as 50 bp. These results demonstrate the potential of CRISPR/Cas9 for precise genome editing in *L. starkeyi*, enabling advanced functional genomics and metabolic engineering aimed at sustainable lipid and oleochemical production.

## Materials and methods

### Micro-organisms and culture conditions

The strains used in this study are listed in Table S1. *Lipomyces starkeyi* CBS1807 was obtained from the Central Office for Mold Cultures (Utrecht, The Netherlands). The HR-proficient mutant strain *L. starkeyi* ∆*lslig4*, derived from CBS1807, has been described previously (Oguro et al. 2017). CBS1807 and ∆*lslig4* were cultured in yeast extract/peptone/dextrose (YPD) medium consisting of 1% yeast extract (Kyokuto, Tokyo, Japan), 2% polypeptone (Nihon Pharmaceutical, Tokyo, Japan), and 2% glucose. The uracil-auxotrophic phenotype of *L. starkeyi* transformants was assessed by culturing the strains on a synthetic defined medium [0.17% yeast nitrogen base without amino acids and ammonium sulfate (Thermo Fisher Scientific, Waltham, MA, USA), supplemented with 0.5% ammonium sulfate (FUJIFILM Wako Pure Chemical, Osaka, Japan), and 2% glucose] with or without uracil. For selection, the medium was supplemented, as required for each experiment, with 100 µg/ml hygromycin B (FUJIFILM Wako Pure Chemical), 30 µg/ml nourseothricin (FUJIFILM Wako Pure Chemical), 100 µg/ml geneticin (GIBCO, NY, USA), and/or 20 mM uracil (FUJIFILM Wako Pure Chemical). Solid media were prepared by adding 2% agar (FUJIFILM Wako Pure Chemical). The construction methods for the strains newly generated in this study are described in the Yeast transformation and gene disruption section below.

The *Escherichia coli* strains HST08 (Takara Bio, Shiga, Japan) and Rosetta2 (DE3) pLysS (Novagen, Madison, WI, USA) were used for plasmid construction and recombinant protein expression, respectively. Cultures were grown at 37°C in L-broth composed of 1% Bacto^TM^ Tryptone (Thermo Fisher Scientific), 0.5% Bacto^TM^ Yeast Extract (Thermo Fisher Scientific), 1% NaCl, and 100 µg/ml of ampicillin (FUJIFILM Wako Pure Chemical).

### General molecular biology techniques

Genomic DNA from *L. starkeyi* cells was isolated as previously described (Oguro et al. 2017). Plasmid DNA was purified using the FastGene^®^ Plasmid Mini Kit (Nippon Genetics, Tokyo, Japan). Polymerase chain reaction (PCR) was performed with KOD FX Neo DNA polymerase (Toyobo, Osaka, Japan) according to the manufacturer’s instructions. PCR products were purified using the FastGene^®^ Gel/PCR extraction kit (Nippon Genetics).

### Plasmid construction

The primers used for plasmid construction are listed in Table S2. The genes *AcGFP* encoding *Aequorea coerulescens* green fluorescent protein (GenBank accession no. AAN41637), and *Cas9SV40*, encoding *Streptococcus pyogenes* Cas9 (GenBank accession no. WP_038 431 314) with the SV40 nuclear localization signal (NLS) (PKKKRKV) fused at its C-terminus, were codon-optimized for *L. starkeyi* and synthesized commercially (GenScript Japan, Tokyo, Japan) (Supplementary Figs. S1 and S2). Codon optimization was performed based on the codon usage bias of *L. starkeyi*, in which infrequently used codons were replaced with preferred synonymous codons to improve translational efficiency.

Plasmid pKS/Cas9SV40OE, designed for Cas9SV40 expression in *L. starkeyi*, contained the *LsTDH3* promoter/*sNAT1*/*LsTDH3* terminator and 70486 promoter/*Cas9SV40*/70 486 terminator cassettes with a portion of the *L. starkeyi* 18S rRNA gene. It was constructed by assembling five DNA fragments using NEBuilder HiFi DNA Assembly Master Mix (New England Biolabs, Ipswich, MA, USA). Two DNA fragments corresponding to the 70 486 promoter and terminator were amplified from *L. starkeyi* CBS1807 genomic DNA using the primer sets 70486p-18S Fw/70486p-Cas9 Rv and 70486t-Cas9 Fw/70486t-sNAT Rv, respectively. The 5′ and 3′ fragments of *Cas9SV40* were amplified from synthetic DNA using the primer sets Cas9N-70486p Fw/Cas9N-C Rv and Cas9C-N Fw/Cas9C-70486t Rv, respectively. The vector backbone fragment was amplified with the primer set sNAT-18S Fw/sNAT-18S Rv using pKS-sNAT (Oguro et al. 2017) as a template.

Plasmid pKS/AcGFPOE, containing the *LsTDH3* promoter/*hph*/*LsTDH3* terminator and 70486 promoter/*AcGFP*/70 486 terminator cassettes together with a portion of the *L. starkeyi* 18S rRNA gene locus for expression in *L. starkey*i, was constructed by assembling four DNA fragments using the NEBuilder HiFi. Two DNA fragments corresponding to the promoter and terminator regions of transcript ID 70486 were amplified from *L. starkeyi* CBS1807 genomic DNA using the primer sets 70486p-V Fw/70486p-GFP Rv and 70486t-AcGFP Fw/70486t-V Rv, respectively. The AcGFP coding sequence was amplified from synthetic DNA using the primer set AcGFP-70486p Fw/AcGFP-70486t Rv. The vector backbone fragment was amplified with the primers Vector-70486t Fw/Vector-70486p Rv using pKS-18S-hph (Oguro et al. 2015) as a template. Transcript ID 70486 was selected as a promoter candidate because RNA-seq data indicated that it is expressed at a high and constitutive level in *L. starkeyi* (Mine et al. 2025).

Plasmid pKS/AcGFP-(promoterless) was constructed by PCR-amplifying two DNA fragments from plasmid pKS/AcGFPOE using the primer sets GFP-V Fw/GFP-V Rv and Vector-GFP Fw/Vector-GFP Rv with the NEBuilder HiFi.

Plasmid pKS/∆LsURA3/3000, designed for targeted disruption of *LsURA3*, contained the 5′ and 3′ flanking regions of *LsURA3* (∼3 kb each, including untranslated regions and partial open reading frame sequences) and an *LsACT1* promoter/*KanR*/*LsACT1* terminator cassette. This plasmid was constructed by assembling four PCR fragments using NEBuilder HiFi. The *LsACT1* promoter/*KanR*/*LsACT*1 terminator fragment was amplified from pKS-KanR-1803 (Mine et al. 2025) using primers KanR-LsURA3 5′ HR3000 Fw/KanR-LsURA3 3′ HR3000 Rv. The 5′ and 3′ homologous arms of *LsURA3* were amplified from *L. starkeyi* CBS1807 genomic DNA using the primers LsURA3 5′ HR3000-V Fw/LsURA3 5′ HR3000-KanR Rv and LsURA3 3′ HR3000-KanR Fw/LsURA3 3′ HR3000-V Rv, respectively. The vector backbone fragment (Vbura) was amplified from pBluescript II KS (+) with primers Vector-LsURA3 3′ HR Fw/Vector-LsURA3 5′ HR Rv. Furthermore, homology arms spanning 250, 500, 1000, and 2000 bp in length, together with the drug resistance marker region, were PCR-amplified from plasmid pKS/∆LsURA3/3000 using the primer sets LsURA3 HR250-V Fw/LsURA3 HR250-V Rv, LsURA3 HR500-V Fw/LsURA3 HR500-V Rv, LsURA3 HR1000-V Fw/LsURA3 HR1000-V Rv, and LsURA3 HR2000-V Fw/LsURA3 HR2000-V Rv, respectively. Each amplified DNA fragment was assembled with the vector backbone fragment (Vbura), originally used to construct pKS/∆LsURA3/3000, using the NEBuilder HiFi, resulting in the plasmids pKS/∆LsURA3/250, pKS/∆LsURA3/500, pKS/∆LsURA3/1000, and pKS/∆LsURA3/2000.

For recombinant expression of Cas9SV40 in *E. coli*, the plasmid pCold-GST/Cas9SV40 was constructed by assembling a fragment amplified from pCold-GST (Takara Bio) with primers Vector-Cas9 Fw/Vector-Cas9 Rv and another amplified from the synthetic *Cas9SV40* DNA with primers Cas9-V Fw/Cas9-V Rv using the NEBuilder HiFi.

### Expression and purification of recombinant Cas9SV40

To express recombinant Cas9SV40 with N-terminal His and GST tags, *E. coli* Rosetta2(DE3)pLysS cells were transformed with the plasmid pCold-GST/Cas9SV40 and precultured overnight at 37°C in L broth supplemented with 100 µg/ml ampicillin. The preculture was inoculated into 50 ml of L broth containing 100 µg/ml ampicillin till the OD600 reached 0.1 and then further cultivated at 37°C until the OD600 reached 0.65. At this point, the culture was rapidly cooled to 20°C and incubated for 30 min without shaking. Protein expression was induced by adding IPTG to a final concentration of 50 µM, followed by shaking incubation at 20°C for 18 h. Cells were harvested by centrifugation (10 000 × g, 5 min, 4°C), resuspended in 5 ml extraction buffer [20 mM Tris-HCl (pH 8.0), 500 mM NaCl, 25 mM imidazole], and disrupted by sonication on ice. After removing cell debris using centrifugation (10 000 × g, 30 min, 4°C), the supernatant was loaded onto a HisTrap HP column (Cytiva, USA). The column was washed with 10 ml extraction buffer, and recombinant His-GST-Cas9SV40 was eluted with elution buffer [20 mM Tris-HCl (pH 8.0), 500 mM NaCl, 250 mM imidazole]. The eluate containing His-GST-Cas9SV40 was incubated with HRV 3C protease (10 U per 1 mg protein; Takara Bio) at 4°C for 16 h to cleave the His-GST tag. Then, Ni-NTA agarose (QIAGEN, Hilden, Germany) was added, and the mixture was incubated at room temperature for 30 min. After centrifugation (500 × g, 10 min), the His-GST tag was retained by the resin, and the flow-through containing recombinant Cas9SV40 was collected.

### Design and preparation of sgRNAs

The sg RNAs targeting *AcGFP* and *LsURA3* were designed using CHOPCHOP v3 (Labun et al. 2019). Transcription templates for sgRNAs(GFP1), sgRNA(GFP2), sgRNA(LsURA3-1), and sgRNA(LsURA3-2) were generated by overlap extension PCR using the following primer sets: sgRNA (GFP1) Fw/sgRNA Rv1, sgRNA (GFP2) Fw/sgRNA Rv1, sgRNA (LsURA3-1) Fw/sgRNA Rv2, and sgRNA (LsURA3-2) Fw/sgRNA Rv2, respectively (Table S3). *In vitro* transcription of the sgRNAs was performed using the Guide-it sgRNA In Vitro Transcription Kit (Takara Bio) according to the manufacturer’s instructions.

### Cas9SV40-sgRNA cleavage assay

The target DNA for Cas9SV40, *AcGFP*, was amplified from pKS/Cas9SV40OE using the primer set GFPtemp Fw/GFPtemp Rv (Table S4), and the PCR product was purified. The sgRNA (100 µg) and recombinant Cas9SV40 (500 µg) were combined and incubated at 37°C for 5 min. The reaction was then supplemented with 5 × cleavage buffer [500 mM Tris-HCl (pH 7.9), 1 M NaCl, 100 mM MgCl_2_, and 10 mM DTT] and nuclease-free water, followed by a 10-min incubation at 37°C. Next, after adding 250 ng of target DNA (*AcGFP*), the reaction was continued at 37°C for 1 h and terminated by incubation at 80°C for 5 min. The products were analyzed by 2% agarose gel electrophoresis. *LsURA3* was amplified using *L. starkeyi* genomic DNA as the template and the LsURA3temp Fw/LsURA3temp Rv primer set (Table S4). This gene was also subjected to the cleavage assay under the same conditions.

### Yeast transformation and gene disruption

For transformation, pKS/Cas9SV40OE was linearized with HpaI and introduced into *L. starkeyi* CBS1807 and ∆*lslig4* via electroporation, resulting in integration at the 18S rRNA gene locus. The resulting transformants were designated as WT/*Cas9* and ∆*lslig4*/*Cas9* strains (Table S1). Furthermore, pKS/AcGFPOE and pKS/AcGFP*-*(promoterless) were digested with ApaI and transformed into the WT/*Cas9* strain via electroporation, also resulting in integration at the 18S rRNA gene locus, yielding the WT/*Cas9*/*GFP* and WT/*Cas9*/*GFP-*(promoterless) strains (Table S1).

To disrupt *AcGFP*, 0.25, 0.5, 1, 3, or 5 µg of sgRNA targeting *AcGFP* was introduced into the WT*/Cas9*/*GFP* strain via electroporation. To disrupt *LsURA3*, 3 µg of sgRNA targeting *LsURA3* and/or 1 µg of linear DNA fragments obtained by BamHI digestion of plasmids pKS/∆LsURA3/250, pKS/∆LsURA3/500, pKS/∆LsURA3/1000, pKS/∆LsURA3/2000, and pKS/∆LsURA3/3000, as well as a PCR-amplified DNA fragment generated using pKS/∆LsURA3/3000 as a template with the primer set LsURA3 HR50-KanR Fw/LsURA3 HR50-KanR Rv (Table S2), were introduced into WT/*Cas9* or ∆*lslig4*/*Cas9* strains via electroporation. The resulting transformants were designated WT/*Cas9*/∆*lsura3* and ∆*lslig4*/*Cas9*/∆*lsura3* strains (Table S1).

*Lipomyces starkeyi* cells were electroporated as previously described (Takaku et al. 2020b). Cells were grown in YPD medium till the mid-logarithmic phase, chilled on ice, and harvested by centrifugation. The pellets were resuspended in 10 ml of buffer (1 mM Tris-HCl, 0.1 mM EDTA, and 0.2 M LiAc, pH 8.0) and incubated at 30°C for 45 min with gentle shaking. Subsequently, after adding 100 µl of 1 M dithiothreitol, the suspension was incubated for an additional 15 min at 30°C with gentle shaking. The cells were washed twice with 50 ml of ice-cold sterile distilled water and once with 3 ml of 0.5 M ice-cold sucrose. After centrifugation, 40 µl of the resulting cell suspension was mixed with sgRNA and/or DNA fragments, incubated on ice for 5 min, and transferred into 0.2-cm electroporation cuvettes. Electroporation was performed at a field strength of 3.75 kV/cm, resistance of 800 Ω, and capacitance of 25 µF. The electroporated cells were immediately mixed with 1 ml of 0.5 M ice-cold sucrose and allowed to recover for 12 h at 30°C with gentle shaking. These cultures were centrifuged, resuspended in sterile distilled water, and plated on YPD agar medium supplemented with 100 µg/ml geneticin or 30 µg/ml nourseothricin for selection.

The correct integration of the constructs in these transformants was confirmed via colony PCR (Fig. S3).

Unless otherwise stated, transformation experiments were independently repeated at least twice, and consistent trends were observed.

### Detection of GFP fluorescence in yeast colonies

GFP fluorescence in yeast colonies grown on solid medium was detected using a ChemiDoc™ Touch MP Imaging System (Bio-Rad, Hercules, CA, USA). Agar plate lids were removed prior to imaging, and colonies were illuminated using the blue epi-illumination mode. Exposure times were adjusted according to fluorescence intensity and set to 0.25 s for the WT/*Cas9*/*GFP*-(promoterless) strain and 20 ms for the WT/*Cas9*/*GFP* strain. Fluorescence images were acquired and processed using the manufacturer’s Image Lab software.

## Results

### Establishment of the Cas9/sgRNA system in *Lipomyces starkeyi*

To establish a functional CRISPR/Cas9 genome-editing system in *L. starkeyi*, we first constructed a Cas9 expression module. Because efficient Cas9 activity requires nuclear localization, *Streptococcus pyogenes Cas9* was fused with the SV40-derived NLS at its C-terminus, which is widely used to ensure the nuclear import and activity of Cas9–sgRNA complexes in diverse yeast species (DiCarlo et al. 2013, Schwartz et al. 2016, Weninger et al. 2018, Otoupal et al. 2019).

To enhance intracellular expression, *Cas9SV40* was codon-optimized for *L. starkeyi* and expressed using a constitutively active promoter derived from transcript ID 70486. The resulting plasmid, pKS/Cas9SV40OE, was linearized and transformed into the wild-type strain CBS1807, where it was integrated into the 18S rRNA gene locus, generating the WT/*Cas9* strain.

Unlike other model yeasts, such as *Saccharomyces cerevisiae*, RNA polymerase III–driven promoters suitable for sgRNA expression, including U6 or SNR52, have not yet been experimentally validated in *L. starkeyi* (DiCarlo et al. 2013, Jiao et al. 2019). Therefore, instead of relying on intracellular sgRNA expression, sgRNAs were synthesized *in vitro* using T7 RNA polymerase and directly introduced into *L. starkeyi* cells. This strategy simplified system design and minimized the variability associated with sgRNA transcription and processing, as similarly reported in other fungal systems employing *in vitro*-transcribed sgRNAs (Liu et al. 2015).

To validate Cas9 functionality *in vivo*, the codon-optimized *Aequorea coerulescens GFP* (*AcGFP*) gene was employed as a fluorescence reporter. pKS/AcGFPOE and pKS/AcGFP-(promoterless) were linearized and transformed into the WT/*Cas9* strain, where the constructs were integrated into the 18S rRNA gene locus, yielding WT/*Cas9*/*GFP* and WT/*Cas9*/*GFP*-(promoterless) strains, respectively. Under blue epi-illumination, only the WT/*Cas9*/*GFP* colonies exhibited strong GFP fluorescence, confirming correct reporter expression and establishing a fluorescence-based readout for subsequent genome-editing analyses (Fig. S4).

### Validation of CRISPR/Cas9 activity and *in vivo* mutation analysis

**Figure 1:**
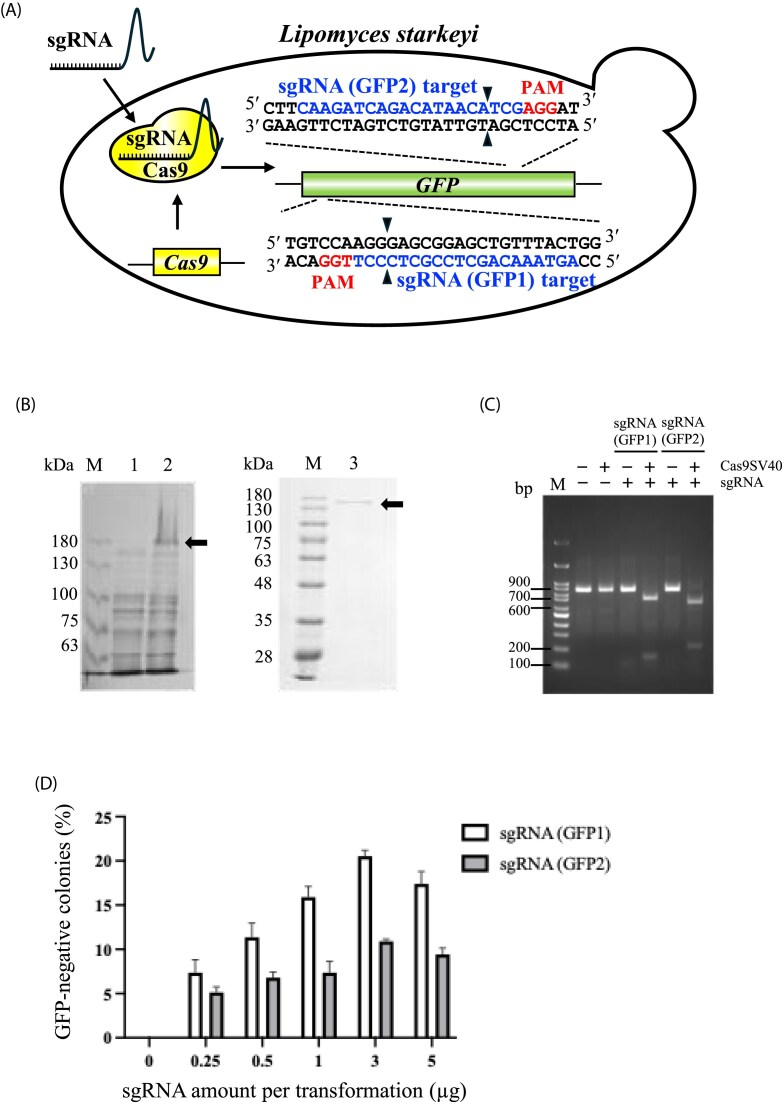

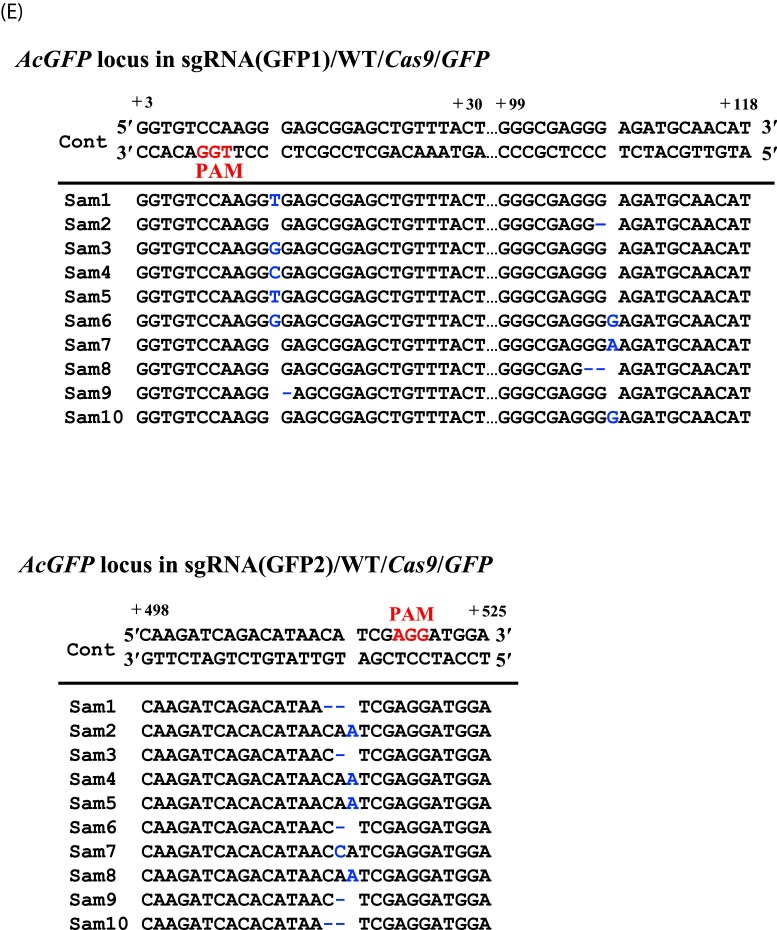
CRISPR/Cas9-mediated genome editing in *L. starkeyi*:(A) Schematic representation of the sgRNAs designed to target the *AcGFP* gene. Two sgRNAs for GFP1 and GFP2 were designed to induce double-strand breaks (DSBs) at specific positions within *AcGFP* for targeted disruption. The sgRNA positions relative to the target loci and their respective cleavage sites are indicated. (B) SDS-PAGE analysis of recombinant Cas9SV40 protein expression. Lane 1: cell extract before induction; Lane 2: cell extract after 18 h of induction at 20°C following initial incubation at 37°C; Lane 3: purified recombinant Cas9SV40 protein, shown as the predominant band of ∼160 kDa. This confirmed the successful expression and purification of Cas9SV40. (C) *In vitro* cleavage assay for *AcGFP* with sgRNA (GFP1) and sgRNA (GFP2) showing successful Cas9SV40-induced DSBs at the expected cleavage sites. (D) Percentage of GFP-negative colonies following transformation with varying concentrations of sgRNAs targeting *AcGFP*. Error bars represent the standard deviation of independent experiments (n = 3). (E) Sequencing analysis of the *AcGFP* locus in sgRNA(GFP1)/WT/*Cas9*/*GFP* and sgRNA(GFP2)/WT/*Cas9*/*GFP* transformants. In sgRNA(GFP1) transformants, mutations were detected at the canonical cleavage site (+13/14) and at a nearby position (+107/108), with five colonies corresponding to each site. In sgRNA(GFP2) transformants, all 10 analyzed colonies exhibited mutations at the predicted cleavage site (+514/515).

Two sgRNAs targeting distinct regions of *AcGFP*, sgRNA(GFP1) and sgRNA(GFP2), were designed using the CHOPCHOP platform (Fig. 1A) (Labun et al. 2019). Details of sgRNA design and preparation are described in the Materials and Methods section. Their cleavage activities were first evaluated *in vitro* using recombinant Cas9SV40 protein. The *Cas9SV40* gene was cloned into the pCold-GST vector, expressed in *E. coli* Rosetta2(DE3)pLysS (Fig. 1B). The recombinant Cas9SV40 protein was then purified following GST tag removal and used for subsequent cleavage assays. SDS-PAGE analysis revealed a dominant band of approximately 160 kDa, consistent with the predicted molecular mass of Cas9SV40 (Fig. 1B).

Both sgRNAs efficiently directed Cas9SV40-mediated cleavage of the *AcGFP* DNA fragment at the expected target sites *in vitro*, confirming their functionality (Fig. 1C). To assess genome editing *in vivo*, increasing amounts of sgRNA(GFP1) or sgRNA(GFP2) were introduced into the WT/*Cas9*/*GFP* strain (Fig. 1A). Loss of GFP fluorescence was readily detected after treatment with ≥ 3 µg of sgRNA, with sgRNA(GFP1) consistently exhibiting higher disruption efficiency (Fig. 1D).

Sequencing analysis of the *AcGFP* locus in non-fluorescent colonies confirmed CRISPR/Cas9-induced frameshift mutations. In the sgRNA(GFP2)/WT/*Cas9*/*GFP* transformants, all 10 analyzed colonies carried mutations at the predicted cleavage site (+514/515) (Fig. 1E). In contrast, sgRNA(GFP1)/WT/*Cas9*/*GFP* transformants exhibited mutations either at the canonical cleavage site (+13/14) or at a nearby position (+107/108), with five colonies corresponding to each site (Fig. 1E). This is consistent with previous reports in *S. cerevisiae*, in which Cas9 displayed high sequence specificity *in vitro* but occasional cleavage at proximal sites *in vivo* (Fu et al. 2016). In *L. starkeyi*, similar effects may arise from local chromatin accessibility or partial complementarity between sgRNA(GFP1) and adjacent *AcGFP* sequences.

Collectively, these results demonstrate that *in vitro* cleavage assays reliably predict *in vivo* sgRNA activity in *L. starkeyi*, confirming that a functional CRISPR/Cas9 genome-editing platform was successfully established in this yeast (Fig. 1A).

### CRISPR/Cas9-mediated enhancement of homologous recombination at the *LsURA3* locus

Due to low targeting efficiency and the requirement for long homologous regions, non-HR predominantly occurs in *L. starkeyi* under standard transformation conditions, limiting targeted gene replacement (Oguro et al. 2017). In addition to inducing sequence-specific double-strand DNA breaks (DSBs), the CRISPR/Cas9 system increase the frequency of HR between genomic DNA and exogenous donor fragments, including cases in which donor DNA contains shortened homologous regions (Stovicek et al. 2017, Zheng et al. 2018). This study aimed to investigate whether CRISPR/Cas9 could enhance HR efficiency at the *LsURA3* locus in *L. starkeyi*.

**Figure 2:**
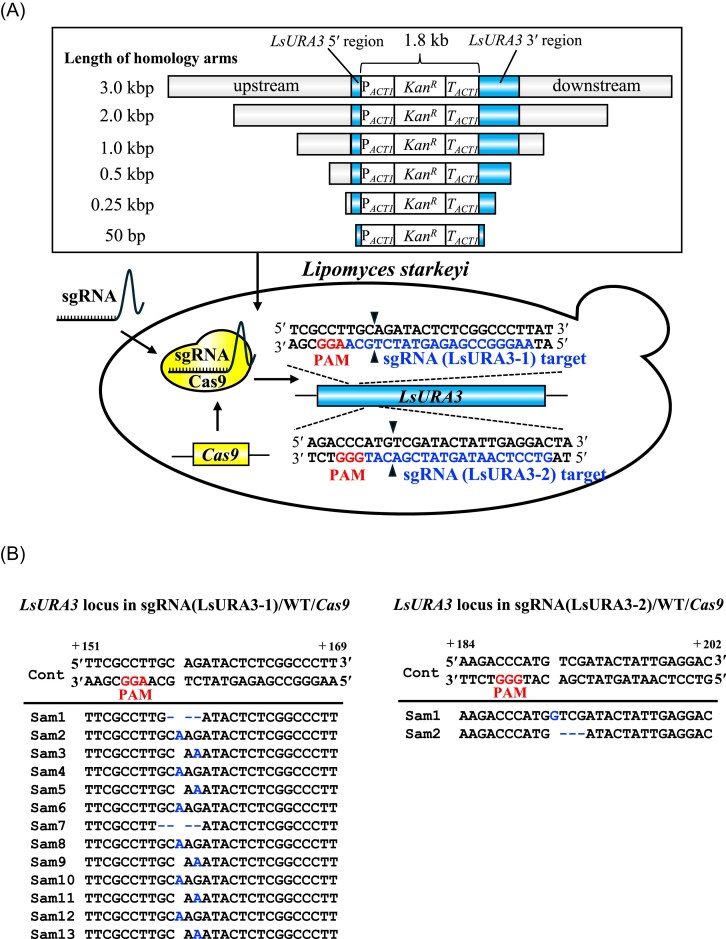
Schematic representation of the CRISPR/Cas9-mediated *LsURA3* gene-targeting strategy and homologous recombination. (A) Schematic representation of the CRISPR/Cas9-mediated *LsURA3* gene-targeting strategy in *L. starkeyi*. Two sgRNAs (LsURA3-1 and LsURA3-2) were designed to induce double-strand breaks (DSBs) at specific loci within *LsURA3*. Homologous regions of varying lengths (50, 250, 500, 1000, 2000, and 3000 bp) were designed to flank the *KanR* cassette for HR at the *LsURA3* locus. The sgRNAs positions relative to the target loci and their respective cleavage sites are also depicted. (B) Sequencing analyses of the *LsURA3* loci in sgRNA(LsURA3-1)/WT/*Cas9* and sgRNA(LsURA3-2)/WT/*Cas9* transformants. In the former, 10/10 uracil-auxotrophic colonies showed frameshift mutations due to insertions or deletions at the predicted cleavage site (+160/161), while in the latter, two uracil-auxotrophic colonies exhibited frameshift mutations near the predicted cleavage site (+193/194) due to insertions or deletions.

The cleavage activities of two sgRNAs (LsURA3-1 and LsURA3-2), designed using the CHOPCHOP tool (Fig. 2A), were evaluated *in vitro* following complex formation with Cas9SV40. Both sgRNAs induced DSBs at the expected target sites, confirming their functionality (Fig. S5).

Experimental procedures for sgRNA delivery and mutant screening are described in the Materials and Methods section. Next, we introduced 3 µg of each sgRNA (LsURA3-1 and LsURA3-2) into the WT/*Cas9* strain by electroporation. We randomly selected 60 transformants from the resulting cultures and plated them on SD medium with or without uracil. The results showed that sgRNA(LsURA3-1)/WT/*Cas9* transformants yielded 21.7% (13/60) uracil-auxotrophic colonies, whereas sgRNA(LsURA3-2)/WT/*Cas9* transformants yielded 3.3% (2/60) uracil-auxotrophic colonies. We further sequenced the *LsURA3* locus of 10 uracil-auxotrophic colonies from sgRNA(LsURA3-1)/WT/*Cas9* and two colonies from sgRNA(LsURA3-2)/WT/*Cas9*. Sequencing results revealed that all 10 colonies from sgRNA(LsURA3-1)/WT/*Cas9* showed frameshift mutations near the expected cleavage site (+160/161) due to insertions or deletions (Fig. 2B), as did the two colonies from sgRNA(LsURA3-2)/WT/*Cas9* (the predicted cleavage site being + 193/194) (Fig. 2B). Based on these findings, we chose to proceed with sgRNA(LsURA3-1) for further experiments aimed at enhancing HR efficiency.

To assess whether CRISPR/Cas9 enhanced HR efficiency, disruption constructs containing homologous regions of varying lengths (50, 250, 500, 1000, 2000, or 3000 bp) were introduced into the WT/*Cas9* strain, either with or without sgRNA(LsURA3-1) (Fig. 2A). The ability to replace the *LsURA3* locus with a *KanR* cassette via HR was then evaluated. Transformants that grew on geneticin- and uracil-containing media were randomly selected (n = 60). To select the uracil-auxotrophic colonies, transformants were grown on SD agar plates with or without uracil, and 30 candidates were selected and further examined by colony PCR to confirm the correct gene replacement.

In the absence of sgRNA (CRISPR/Cas9-independent conditions), transformation with constructs containing homologous regions ≤1000 bp did not produce any uracil-auxotrophic strains, while those with 2000- and 3000-bp homologous regions yielded uracil-auxotrophic strains at frequencies of 6.7% (4/60) and 13.3% (8/60), respectively. Colony PCR analysis confirmed precise gene replacement in all candidates with 2000-bp homology and in 87.5% (7/8) in those with 3000-bp homology (Table 1).

**Table 1:** Enhanced CRISPR/Cas9-mediated homologous recombination efficiency in ∆*lslig4* compared to WT in *L. starkeyi*.

Strain	Homologous region length (bp)	sgRNA (LsURA3-1)	*LsURA3* Gene disruption efficiency (uracil auxotrophy/colonies)	Homologous recombination efficiency
WT/*Cas9*	50	−	0%(0/60)	−
	250		0%(0/60)	−
	500		0%(0/60)	−
	1000		0%(0/60)	−
	2000		6.7%(4/60)	100% (4/4)
	3000		13.3%(8/60)	87.5% (7/8)
	50	+	83.3%(50/60)	36.0%(11/30)
	250		86.7%(52/60)	40.0%(12/30)
	500		86.7%(52/60)	43.3%(13/30)
	1000		86.7%(52/60)	60.0%(18/30)
	2000		88.3%(53/60)	73.3%(22/30)
	3000		96.7%(58/60)	80.0%(24/30)
∆*lslig4*/*Cas9*	50	−	0%(0/60)	−
	250		0%(0/60)	−
	500		31.7%(19/60)	100%(19/19)
	1000		83.3%(50/60)	100%(30/30)
	2000		91.7%(55/60)	100%(30/30)
	3000		95%(57/60)	100%(30/30)
	50	+	100%(60/60)	100%(30/30)
	250		100%(60/60)	100%(30/30)
	500		100%(60/60)	100%(30/30)
	1000		100%(60/60)	100%(30/30)
	2000		100%(60/60)	100%(30/30)
	3000		98.3%(59/60)	100%(30/30)

In the presence of sgRNA (CRISPR/Cas9-dependent conditions), uracil-auxotrophic strains were obtained at a high frequency even with 50-bp homologous regions (83.3%, 50/60). Although the frequency increased to 96.7% (58/60) with 3000-bp homologous regions, no substantial differences were observed among constructs containing 50–2000 bp-long homology arms. However, colony PCR analysis revealed that precise gene replacement occurred in only 36% (11/30) of colonies generated using constructs with 50-bp homologous regions. These results indicate that the accuracy of gene replacement increased with the length of the homologous region, reaching 80% (24/30) with 3000-bp homology (Table 1).

Together, these results indicate that CRISPR/Cas9-induced DSBs substantially enhance the frequency of HR at the *LsURA3* locus in *L. starkeyi*, whereas the accuracy of gene replacement in the WT/*Cas9* background remains dependent on the length of the homologous region. Similar trends in homologous recombination efficiency and replacement accuracy were observed across independent experiments.

### Suppression of NHEJ markedly improves HR efficiency in an *LsLIG4* deletion strain

Previous studies have demonstrated that NHEJ suppression can markedly enhance HR efficiency in nonconventional yeasts. In *Yarrowia lipolytica*, deletion of core NHEJ factors, such as *KU70*, promoted targeted genome modification (Schwartz et al. 2016). More directly, in *L. starkeyi*, deletion of *LsLIG4*, which encodes DNA ligase IV responsible for the final ligation step of NHEJ, resulted in a substantial increase in HR-mediated gene-targeting efficiency (Oguro et al. 2017). Based on these findings, to further enhance HR efficiency, the host strain was replaced with an *LsLIG4* deletion strain (Δ*lslig4*/*Cas9*) to suppress NHEJ, and was subjected to the same transformation experiments.

In the absence of sgRNA, uracil-auxotrophic strains were not obtained using constructs with homologous regions ≤250 bp. In contrast, uracil-auxotrophic strains were recovered at frequencies of 31.7% (19/60) with 500-bp homology, 83.3% (50/60) with 1000-bp homology, and > 90% with homologous regions ≥2000 bp (Table 1). Colony PCR analysis confirmed precise gene replacement at the target locus in all examined candidates.

Under CRISPR/Cas9-dependent conditions, uracil-auxotrophic strains were obtained at 100% frequency (60/60) even when homologous regions were below 50 bp (Table 1). Colony PCR analysis further confirmed that all candidates exhibited accurate gene replacement at the target locus.

These results suggest that *LsLIG4* deletion markedly enhances HR efficiency in *L. starkeyi*. Furthermore, under CRISPR/Cas9-dependent conditions, precise gene replacement was achieved with 100% accuracy using minimal homologous regions, consistent with the idea that suppression of NHEJ shifts DSB repair toward HR. Similar trends were observed across independent experiments.

## Discussion

### Establishment of a functional CRISPR/Cas9 platform in *L. starkeyi*

Nonconventional yeasts are being increasingly exploited as industrial hosts, yet their genetic engineering remains constrained by limited, species-validated toolkits and DNA repair landscapes that hinder precise integration. Recent reviews emphasize that many nonconventional yeasts exhibit low HR activity, dependence on NHEJ, and a limited repertoire of well-characterized regulatory elements, collectively compromising the predictability of genome engineering (Park et al. 2022). Therefore, functional genomics and metabolic engineering of *L. starkeyi* remain limited, despite its high potential as an oleaginous yeast.

sgRNA expression is a major practical bottleneck for CRISPR/Cas9 implementation. While Pol III promoters (e.g. U6/SNR52) are routinely used in model yeasts, they are not universally validated across nonconventional species, making promoter engineering a substantial development task (Park et al. 2022). To bypass this dependency, we adopted a Pol III–independent strategy by directly introducing *in vitro*-transcribed sgRNAs into *L. starkeyi*, avoiding uncertainties arising from promoter strength and RNA processing. This approach provides a simple route to establish CRISPR activity in species where Pol III regulatory parts remain understudied.

To enable rapid visual assessment of CRISPR/Cas9 activity, we used a GFP disruption assay, in which editing efficiency was determined based on the loss of GFP signal in yeast colonies. This system represents a convenient, scalable readout of mutagenesis for yeast (Zhou et al. 2024). Together, a flexible CRISPR/Cas9 platform was developed in *L. starkeyi* that is independent of endogenous Pol III promoters and supports straightforward sgRNA validation.

### CRISPR/Cas9-induced DSBs and competition between NHEJ and HR in *L. starkeyi*

CRISPR/Cas9 editing outcomes are shaped by how cells repair Cas9-induced DSBs, primarily through NHEJ or HR. In organisms where NHEJ is dominant, DSBs frequently resolve through end joining, which can elevate mutant recovery while limiting precise donor-directed replacement unless HR is strongly favored (Liao et al. 2024).

In the WT/*Cas9* background of *L. starkeyi*, DSB induction substantially increased the recovery of uracil-auxotrophic transformants even with short-homology arms, indicating that the overall frequency of repair events was enhanced. However, accurate gene replacement remained strongly dependent on homology arm length, with longer arms yielding markedly higher rates of correct replacement. This pattern is consistent with a repair competition model in which DSBs were mostly repaired via NHEJ when homology is limited, whereas longer homology arms more effectively engage HR and stabilize accurate donor-mediated repair (Liao et al. 2024).

Thus, in *L. starkeyi*, Cas9 cutting alone increases the likelihood of repair outcomes at the target locus, but achieving high-precision replacement with minimal homology requires shifting repair-pathway choice toward HR.

### NHEJ suppression enables efficient and precise genome editing in *L. starkeyi*

Among nonconventional yeasts, genetic reduction of NHEJ is a widely-used strategy for precise gene targeting. This is usually accomplished by disabling core components such as Ku or Lig4, thereby increasing the proportion of HR-mediated outcomes among surviving transformants. Importantly, *L. starkeyi* provides a species-specific precedent: deletion of *LsLIG4* (DNA ligase IV) was reported to markedly increase gene-targeting efficiency, supporting NHEJ reduction as a rational route to improve HR in this yeast (Jia et al. 2025).

Consistent with this framework, *LsLIG4* deletion in our study profoundly altered editing outcomes. In the Δ*lslig4*/*Cas9* strain, precise gene replacement was achieved with complete accuracy using homology arms as short as 50 bp under CRISPR/Cas9-dependent conditions. Contrastingly, in the WT/Cas9 background, short-homology constructs generated many candidates but a smaller fraction of correct replacements. Collectively, these results indicate that NHEJ suppression effectively redirects DSB repair toward HR in *L. starkeyi*, enabling highly accurate genome editing with minimal donor homology requirements.

From a genetic engineering perspective, short-homology replacement reduces construct burden and accelerates iterative strain development, facilitating pathway refactoring and high-throughput functional genomics in oleaginous yeasts (Park et al. 2022).

Although the enhanced HR phenotype observed in the Δ*lslig4* background is consistent with the known role of DNA ligase IV in NHEJ, complementation analysis was not performed in the present study. Therefore, the contribution of *LsLIG4* loss to this phenotype should be interpreted cautiously and warrants further validation in future work.

### Implications and future perspectives

The platform described here expands the experimental toolkit for *L. starkeyi* by combining Pol III–independent sgRNA delivery with a rapid reporter readout and a repair-pathway background that supports precise replacement. As recombination engineering is considered a promising route to scalable genome editing in nonconventional yeasts, these design principles can be applied to other industrial species with similar limitations (Park et al. 2022). Future work should aim to integrate this platform with emerging CRISPR modalities and systematically optimize donor design to broaden the scope of precise engineering in oleaginous yeasts (Liao et al. 2024).

## Supplementary Material

foag014_Supplemental_File

## Data Availability

All data generated or analyzed during this study are included in this published article and its Supplementary material files.
