# Supplementary material for: A CRISPR/Cas9-based genome-editing platform enabling efficient and precise gene replacement in Lipomyces starkeyi
Source: FEMS Yeast Res. 2026 Apr 23;26:foag014. doi: 10.1093/femsyr/foag014 (PMC13159722; doi:10.1093/femsyr/foag014)
Supplement: foag014_Supplemental_File [file foag014_supplemental_file.docx]

**Supplementary data**

**A CRISPR/Cas9-based genome-editing platform enabling efficient and precise gene replacement in *Lipomyces starkeyi***

Rikako Sato^a^, Kaito Maruyama^a^, Satoshi Ara^b^, Masayuki Shibata^b^, Yosuke Shida^c^, Wataru Ogasawara^c^, Harutake Yamazaki^a^ Hiroaki Takaku^a^*

*^a^Department of Applied Life Sciences, Niigata University of Pharmacy and Medical and Life Sciences, 265-1 Higashijima, Akiha-ku, Niigata 956-8603, Japan*

*^b^Research Institute for Creating the Future, FUJI OIL Co., Ltd, 4-3 Kinunodai, Tsukubamirai-shi, Ibaraki 300-2497, Japan*

*^c^Department of Bioengineering, Nagaoka University of Technology, 1603-1 Kamitomioka, Nagaoka, Niigata 940-2188, Japan*

*Corresponding author

Phone: +81 250 25 5119

Fax: +81 250 25 5021

E-mail address: htakaku@nupals.ac.jp (H. Takaku)

**Supplementary Table**

**Table S1 Microorganism strains used in this study**

| Strain name | Relevant genotype | Parental strain | Source/references |
| --- | --- | --- | --- |
| *Lipomyces starkeyi*CBS1807 |  |  | Centraalbureau voor Schimmelcultures |
| Δ*lslig4* | *lslig4* ::P_TDH3_-*Sh ble-*T*_TDH3_* | CBS1807 | (Oguro et al., 2017) |
| WT/*Cas9* | 18S rDNA::P*_70486_*-*Cas9SV40*-T*_70486_*- P*_TDH3_*-s*NAT1*-T*_TDH3_* | CBS1807 | This study |
| WT/*Cas9*/*GFP* | 18S rDNA::P*_70486_*-*Cas9SV40*-T*_70486_*- P*_TDH3_*-s*NAT1*-T*_TDH3_* ,  18S rDNA::P*_70486_*-*AcGFP1*-T*_70486_*-P*_TDH3_*-*hph*-T*_TDH3_* | WT/*Cas9* | This study |
| WT/*Cas9*/*GFP-*(promoterless) | 18S rDNA::P*_70486_*-*Cas9SV40*-T*_70486_*- P*_TDH3_*-s*NAT1*-T*_TDH3_* ,  18S rDNA:: *AcGFP1*- P*_TDH3_*-*hph*-T*_TDH3_* | WT/*Cas9* | This study |
| Δ*lslig4*/*Cas9* | *lslig4*::P*_TDH3_*-*sh ble*-T*_TDH3,_*  18S rDNA::P*_70486_*-*Cas9SV40*-T*_70486_*- P*_TDH3_*-s*NAT1*-T*_TDH3_* | Δ*lslig4* | This study |
| WT/*Cas9*/Δ*lsura3* | 18S rDNA::P*_70486_*-*Cas9SV40*-T*_70486_*- P*_TDH3_*-s*NAT1*-T*_TDH3_, lsura3*::P*_ACT1_*-*KanR*-T*_ACT1_* | WT/*Cas9* | This study |
| Δ*lslig4*/*Cas9*/Δ*lsura3* | *lslig4*::P*_TDH3_*-*Sh ble*-T*_TDH3_*, 18S rDNA::P*_70486_*-*Cas9SV40*-T*_70486_*- P*_TDH3_*-s*NAT1*-T*_TDH3_, lsura3*::P*_ACT1_*-*KanR*-T*_ACT1_* | Δ*lslig4*/*Cas9* | This study |
| *Escherichia coli* HST08 | F^－^*, endA1, supE44, thi-1, recA1, relA1, gyrA96, phoA,* Φ80d*lacZ*ΔM15*,* Δ*(lacZYA-argF)*U169*,* Δ*(mrr-hsdRMS-mcrBC), ΔmcrA, λ*^－^ |  | Takara Bio |
| *Escherichia coli* Rosetta2(DE3)pLysS | F^-^*ompT hsdS*_B_(r_B_^-^ m_B_^-^) *gal dcm* (DE3) pLysSRARE2 (Cam^R^) |  | Novagen |

**Table S2 Primers used for plasmid construction in this study**

| Primer name | Primer sequence (5′-3′) |
| --- | --- |
| 70486p-18S Fw | CCGGCTAGTTCTCGCTCTACTCTACTCTAGTCTAAC |
| 70486p-Cas9 Rv | TCTTATCCATTGTGAAAGTTGAATATAGATAGTAAGAGCTTTTTCG |
| 70486t-Cas9 Fw | AAAGGTTTAAACGTCGTCTCGCTTCCTC |
| 70486t-sNAT Rv | AGCAAATTAAGGGAAGAGCAGGGTGGGC |
| Cas9N-70486p Fw | AACTTTCACAATGGATAAGAAGTACAGTATTGGCTTGG |
| Cas9N-C Rv | TGAAGTTACGGTTAGCGAAACCGTCGGAC |
| Cas9C-N Fw | TTTCGCTAACCGTAACTTCATGCAGTTGATCCAC |
| Cas9C-70486t Rv | GAGACGACGTTTAAACCTTTCTCTTCTTCTTAGGGTCC |
| sNAT-18S Fw | TGCTCTTCCCTTAATTTGCTGAAGCGGTTTGCC |
| sNAT-18S Rv | GTAGAGCGAGAACTAGCCGGTACCTTTCCG |
| 70486p-V Fw | GTACCGGCTAGTTCTCGCTCTACTCTACTCTAGTCTAAC |
| 70486p-GFP Rv | CCTTGGACACCATTGTGAAAGTTGAATATAGATAGTAAGAGCTTTTTCG |
| 70486t-AcGFP Fw | CTCTATAAATAAACGTCGTCTCGCTTCCTCTC |
| 70486t-V Rv | TCAGCAAATTAAGGGAAGAGCAGGGTGGGC |
| AcGFP-70486p Fw | ATCTATATTCAACTTTCACAATGGTGTCCAAGGGAGCG |
| AcGFP-70486t Rv | GAGAGGAAGCGAGACGACGTTTATTTATAGAGTTCGTCCATACCGTGTG |
| Vector-70486t Fw | CCCTGCTCTTCCCTTAATTTGCTGAAGCGGTTTGCC |
| Vector-70486p Rv | GAGTAGAGCGAGAACTAGCCGGTACCTTTCCG |
| GFP-V Fw | CGGAAAGGTACCGGCTAGTTATGGTGTCCAAGGGAGCG |
| GFP-V Rv | AAACCGCTTCAGCAAATTAATTATTTATAGAGTTCGTCCATACCGTGTG |
| Vector-GFP Fw | TTAATTTGCTGAAGCGGTTTGCC |
| Vector-GFP Rv | AACTAGCCGGTACCTTTCCG |
| KanR-LsURA3 5′HR3000 Fw | ACGAATTACTTCGTTACCGTCTACCGCTGACG |
| KanR-LsURA3 3′HR3000 Rv | AGTCCTCAATAGAGGAGTATAGAGTTGAATTTAATGGACGTTG |
| LsURA3 5′HR3000-V Fw | GCTTGATGGGCCCGTTAACGGATCCAGTGAATGTGGAGAAGCCG |
| LsURA3 5′HR3000-KanR Rv | GGTAGACGGTAACGAAGTAATTCGTCTTTAGTGTCCAC |
| LsURA3 3′HR3000-KanR Fw | ACTCTATACTCCTCTATTGAGGACTATACATATGAATCAGTCATTGTC |
| LsURA3 3′HR3000-V Rv | CGGGCTGCAGGAATTCGATGGGCCCTCACTGCGATCACTTGATTGTG |
| Vector-LsURA3 3′HR Fw | GGGCCCATCGAATTCCTG |
| Vector-LsURA3 5′HR Rv | GGATCCGTTAACGGGCCC |
| LsURA3 HR250-V Fw | GCTTGATGGGCCCGTTAACGGATCCAATCACAGGCGACAAAAAAATTCACTC |
| LsURA3 HR250-V Rw | CGGGCTGCAGGAATTCGATGGGCCCAAGAGTCCCCTCTCTTCGC |
| LsURA3 HR500-V Fw | GTTAACGGATCCGTATCTGTGTTGTTATTCCTCCGG |
| LsURA3 HR500-V Rw | TTCGATGGGCCCGACAATAATAATGTCACTGCCGCC |
| LsURA3 HR1000-V Fw | GTTAACGGATCCGGTCAACAATGTCGTGCC |
| LsURA3 HR1000-V Rw | TTCGATGGGCCCTGGAAGCGAGCACATCATTAAG |
| LsURA3 HR2000-V Fw | GCTTGATGGGCCCGTTAACGGATCCAATATTACAAGCTCTTATTATTCCACAATTCCTATCCG |
| LsURA3 HR2000-V Rw | CGGGCTGCAGGAATTCGATGGGCCCGTTCCTTGGTGCTGGCGC |
| Vector-Cas9 Fw | AAAGGTTTAACTGCAGTCTAGATAGGTAATCTCTGCTTAAAAGCAC |
| Vector-Cas9 Rv | TCTTATCCATATGTCCCGGGCCCTGGAAC |
| Cas9-V Fw | CCCGGGACATATGGATAAGAAGTACAGTATTGGCTTGG |
| Cas9-V Rv | TAGACTGCAGTTAAACCTTTCTCTTCTTCTTAGGGTCC |
| LsURA3 HR50-KanR Fw | ACGAACCTTTGTGTCTCGCTGGACGTGGACACTAAAGACGAATTACTTCGTTACCGTCTACCGCTGACG |
| LsURA3 HR50-KanR Rv | AAGCTCGACCAGTGGGACAATGACTGATTCATATGTATAGTCCTCAATAGAGGAGTATAGAGTTGAAT |

**Table S3 Primers used for sgRNA template amplification in this study**

| Primer name | Primer sequence (5′-3′) |
| --- | --- |
| sgRNA (GFP1) Fw | CCTCTAATACGACTCACTATAGGAGTAAACAGCTCCGCTCCCTGTTTTAGAGCTAGAAATAGCAAGTTA |
| sgRNA (GFP2) Fw | CCTCTAATACGACTCACTATAGGCAAGATCAGACATAACATCGGTTTTAGAGCTAGAAATAGCAAGTTA |
| sgRNA Rv1 | GCACCACCGACTCGGTGCCACTTTTTCAAGTTGATAACGGACTAGCCTTATTTTAACTTGCTATTTCTAG |
| sgRNA (LsURA3-1) Fw | CCTCTAATACGACTCACTATAGGAAGGGCCGAGAGTATCTGCAGTTTAAGAGCTATGC |
| sgRNA (LsURA3-2) Fw | CCTCTAATACGACTCACTATAGGTCCTCAATAGTATCGACATGTTTAAGAGCTATGC |
| sgRNA Rv2 | AAAAAAAGCACCGACTCGGTGCCACTTTTTCAAGTTGATAACGGACTAGCCTTATTTAAACTTGCTATGCTGTTTCCAGCATAGCTCTTAAAC |

**Table S4 Primers for target DNA amplification in Cas9SV40 cleavage assay**

| Primer name | Primer sequence (5′-3′) |
| --- | --- |
| GFPtemp Fw | GGCTATATATTCCCGGTCC |
| GFPtemp Rv | TTATTTATAGAGTTCGTCCATACCGTGTG |
| LsURA3temp Fw | GGCACTATAGCTCGTACAAGC |
| LsURA3temp Rv | GATCTTGAAGATGCCTGACG |

**Supplementary Figures**

**Figure S1 Sequence of original and optimized *AcGFP* gene** Upper row: the original *AcGFP* sequence (AcGFPori), lower row: the optimized *AcGFP* sequence (AcGFPopt), identical nucleic acids are indicated by asterisks.

**Figure S2 Sequence of original and optimized *Cas9SV40* gene** Upper row: the original *Cas9SV40* sequence (Cas9SV40ori), lower row: the optimized *Cas9SV40* sequence (Cas9SV40opt), identical nucleic acids are indicated by asterisks.

**(A)**

**(B)**

**(C)**

**(D)**

**(E)**

**(F)**

**Figure S3 PCR analysis for confirmation of the genetic transformation** (A) Strategy for verifying integration of the *Cas9SV40* gene into the 18S rDNA locus in *L. starkeyi* CBS1807 (WT) and ∆*lslig4* strains. (B) PCR confirmation of genetic transformation using the strategy outline in (A). Cells were suspended with 100 µL of lysing buffer (10 mM Tris-HCl, 1 mM EDTA, 100 mM NaCl, 2% Triton X-100, 1% SDS, pH 8.0) and incubated for 30 min at 70°C. Following extraction with 100 µL of phenol-chloroform and centrifugation, the supernatant was used as the PCR template. PCR was performed with primer set 18SoutFw (5′-CAGTGAAACTGCGAATGG-3′)/sNat Rv (5′-AGTACGAGACGACCACGAAGC-3′) for 18S rDNA 5′ upstream region (PCR1) and sNatFw (5′-ATGGGTACCACTCTTGACGACAC-3′)/18SoutRv (5′-ACGGTATCTGCGTTAACC-3′) for 18S rDNA 3′ downstream region (PCR2). Correct recombination in the 5′ region (PCR1) yielded a 7.9-kb product, and correct recombination in the 3′ region (PCR2) yielded a 1.9-kb product. Dashed lines indicate removal of superfluous lanes. (C) Strategy for verifying integration of the *AcGFP* gene into the 18S rDNA locus of the WT/*Cas9* strain. (D) PCR confirmation of genetic transformation using the strategy outlined in (C). PCR templates were prepared as described in (B). PCR was performed with primer set 18SoutFw/HygRv (5′-GGTCGGCATCTACTCTATTC-3′) for the 18S rDNA 5′ upstream region (PCR1) and HygFw (5′-ATGAAAAAGCCTGAACTCAC-3′)/18SoutRv for the 18S rDNA 3′ downstream region (PCR2). Correct recombination yielded a 5.0-kb product in the 5′ region (PCR1) and a 2.4-kb product in the 3′ region (PCR2). (E) Strategy for verifying integration of the *KanR* gene into the *LsURA3* locus in WT/*Cas9* and ∆*lslig4*/*Cas9* strains. (F) PCR confirmation of genetic transformation using the strategy outlined in (E). PCR templates were prepared as described in (B). PCR was performed with primer set LsURA3 5′outFw (5′-ACTCCAGCAATGTCTGAGCC-3′)/ LsURA3 3′outRv (5′-GGTAGAATCCTCTGAACATCGG-3′) spanning the *LsURA3* locus. Correct recombination yielded an 8.0-kb PCR product.

**Figure S4 Comparison of GFP Fluorescence in Colonies Induced by Blue Epi Illumination on Agar Plates** Left: Colonies on the agar plate under white light illumination, showing the natural colony morphology. Right: Colonies after Blue epi illumination, showing GFP fluorescence induction. GFP-expressing colonies are highlighted by fluorescence after excitation. Colonies on the agar plate were exposed to Blue epi illumination for different durations: 1/4s for the WT/*Cas9*/*GFP*-(promoterless) strain and 20ms for the WT/*Cas9*/*GFP* strain. GFP fluorescence was induced in colonies of the WT/*Cas9*/*GFP* strain, while no fluorescence was observed in the WT/*Cas9*/*GFP*-(promoterless) strain after the respective illumination times.

**Figure S5 *In vitro* cleavage assay for *LsURA3* with sgRNA (LsURA3-1) and sgRNA (LsURA3-2)**

*In vitro* cleavage assay targeting *LsURA3* using sgRNA (LsURA3-1) and sgRNA (LsURA3-2), demonstrating successful Cas9SV40-induced double-strand breaks (DSBs). The expected cleavage patterns were observed, with the presence or absence of cleavage indicated by the conditions shown at the top. M denotes the DNA marker, and the bp markers on the left indicate the size of the cleavage products. The bands corresponding to the cleavage products show the expected shift, confirming the efficacy of the CRISPR-Cas9 cleavage.
